# Depression among Bangladeshi diabetic patients: a cross-sectional, systematic review, and meta-analysis study

**DOI:** 10.1186/s12888-023-04845-2

**Published:** 2023-05-26

**Authors:** Firoj Al-Mamun, Mahmudul Hasan, Shalini Quadros, Mark Mohan Kaggwa, Mahfuza Mubarak, Md. Tajuddin Sikder, Md. Shakhaoat Hossain, Mohammad Muhit, Mst. Sabrina Moonajilin, David Gozal, Mohammed A. Mamun

**Affiliations:** 1 Department of Public Health and Informatics, Jahangirnagar University, Savar, Dhaka, Bangladesh; 2 CHINTA Research Bangladesh, Savar, Dhaka, Bangladesh; 3 Department of Public Health, University of South Asia, Dhaka, Bangladesh; 4 Department of Biochemistry and Molecular Biology, University of Rajshahi, Rajshahi, Bangladesh; 5 Department of Occupational Therapy, Manipal College of Health Professions (MCHP), Manipal Academy of Higher Education (MAHE), Manipal, 576104 India; 6 Department of Psychiatry, Faculty of Medicine, Mbarara University of Science and Technology, Mbarara, Uganda; 7 Department of Psychiatry and Behavioural Neurosciences, McMaster University Hamilton, Hamilton, ON Canada; 8 Department of Child Health and the Child Health Research Institute, The University of Missouri School of Medicine, Columbia, MO USA

**Keywords:** Depression, Diabetes, Diabetes mellitus, Mental health, Bangladesh, Systematic review

## Abstract

**Aim:**

This study aims to assess the prevalence and associated factors of depression among diabetic patients in a cross-sectional sample and perform a systematic review and meta-analysis of the extant studies to date.

**Methods:**

A face-to-face semi-structured interview of established diabetic patients was conducted in four districts of Bangladesh between May 24 to June 24, 2022, and the Patient Health Questionnaire (PHQ–2) was used to detect depression. PRISMA guidelines were followed to conduct a systematic review and meta-analysis, with Bangladeshi articles published until 3rd February 2023.

**Results:**

The prevalence of depression among 390 diabetic patients was 25.9%. Having secondary education and using both insulin and medication increased the likelihood of depression, whereas being a business professional and being physically active reduced the likelihood of depression. The systematic review and meta-analysis indicated that the pooled estimated prevalence of depression was 42% (95% CI 32–52%). Females had a 1.12-times higher risk of depression than males (OR = 1.12, 95% CI: 0.99 to 1.25, *p* < 0.001).

**Conclusions:**

Two-fifths of diabetic patients were depressed, with females at higher risk. Since depression among diabetic patients increases adverse outcomes, improved awareness and screening methods should be implemented to detect and treat depression in diabetic patients.

**Supplementary Information:**

The online version contains supplementary material available at 10.1186/s12888-023-04845-2.

## Introduction

Diabetes is a chronic and prevalent condition worldwide [1]. In 2017, it was estimated that nearly 500 million individuals were affected by type 2 diabetes, i.e., > 6% of the world’s population, reaching up to > 20% of 70-year-old people [1]. In addition to being the ninth most prominent cause of death, with 1.5 million deaths/year [2], diabetes is a major risk factor for blindness, cardiovascular disease, stroke, renal insufficiency, vascular deficits leading to limb amputation, and many other adverse consequences. According to the report by the International Diabetes Federation, 13,136 for every 100,000 Bangladeshi adults were estimated to have diabetes in 2021, a prevalence that is expected to nearly double by 2045, while also causing more than 75,000 deaths in 2021 alone [3]. Diabetes-related health expenditures exceeded 1 billion USD in 2021, and such a unique healthcare cost burden is expected to continue increasing over time [3].

Based on the American Psychiatric Association Diagnostic and Statistical Manual of Mental Disorders (DSM-5), depression is a mood disorder that manifests as a cluster of symptoms that alter the ability of the individual to function [4]. Cumulative epidemiological evidence indicates a strong, independent, and severity-dependent association between the various stages of diabetes and the prevalence of depression [5]. Although the causal mechanisms underlying this association remain unclear, multiple studies summarized in a systematic review and meta-analysis reported a 1.41 relative risk of developing depression among diabetes mellitus patients [6]. Similarly, the prevalence of depression among type 1 diabetic patients was even higher than in type 2 diabetes, probably illustrating the dampening effect of age and cardiovascular disease as confounders in type 2 diabetes [5, 7]. Several studies have been conducted in Bangladesh, but there are disparities in the prevalence rates of depression reported in the context of diabetes. For example, the prevalence of depression was 61.9% among patients with type-2 diabetes [8], whereas it was 16.5% in another study [9]. Such discrepant depression prevalence rates in the Bangladeshi studies among diabetic patients prompted us to conduct a systematic review of the existing published studies while also performing a cross-sectional survey of depression among Bangladeshi diabetic patients.

## Methods

### Cross-sectional study

#### Study design and population

This study was an exploratory, cross-sectional analytical study that collected data through face-to-face semi-structured interviews. Data were collected between May 24, 2022, and June 24, 2022. The study population of this study consisted of general diabetic patients seeking treatment in below mentioned diabetic centers. The study followed the STROBE checklist guidelines.

Out of 64 districts in Bangladesh, four conveniently selected districts were surveyed and assumed to be representative of the rest of the country: Dhaka, Gazipur, Sirajganj, and Kurigram. These regions included both hospitals or specialized diabetes centers as follows: Dhaka Medical College Hospital, BIRDEM General Hospital, Dhaka Central International Medical College Hospital, North Bengal Medical College Hospital, Al Madina Hospital and Diabetic Center, Galaxy Hospital, Gazipur Diabetic Center, Maowna Diabetic Center, Kapasiya Diabetes Somety, Konabari Popular Hospital, and Nageswari Diabetic Center.

#### Inclusion and exclusion criteria

This study was conducted among individuals with a previous diagnosis of diabetes. For inclusion in this study, participants were identified and enrolled if (i) they were present at the facilities in the context of diabetes-related treatment, (ii) were suffering from any sub-type of diabetes (e.g., type I and type II, or gestational diabetes), (iii) were physically, and mentally capable of participating in the study. In addition, the exclusion criteria were individuals with diabetes attending the clinics who were < 18 years of age.

#### Sample size and sampling technique

The non-probability sampling technique was used for this study. Both districts and hospitals were specifically selected to recruit diabetic patients. Before data collection, a research team of 7 people was trained on the study aims and methodology. The sample size was calculated based on a previous study with a prevalence of 40.5% [10], a margin of error of 5%, with a 95% of confidence interval; the estimated sample size was 371. A total of 400 diabetic patients were identified as fulfilling inclusion criteria and were approached for enrollment and interview. However, four subjects declined to participate; 390 complete datasets were collected (6 failed to provide sufficient information) and used for final analysis.

#### Measures

##### Socio-demographic information

Socio-demographic information such as age, gender, marital status, current residence, educational attainment, monthly family income, occupation, presence of chronic diseases, and smoking were included in the survey. In addition, information about physical activities was explored, with an average of at least 30 min of physical activities every day being considered active. The variables, age, educational qualification, and monthly family income were open-ended questions, whereas the rest were close-ended.

##### Diabetic profile

First, participants were asked if anyone had diabetes in their families, such as parents and grandparents. The duration of subject diabetes in years and the type of diabetes were also inquired. Questions on how much time subjects visited the physician for treatment of diabetes in the last six months and medications used to control diabetes were enumerated based on (i) only medication use, (ii) only insulin use, (iii) both medication and insulin use, and (iv) no medication and no insulin.

##### Patient health questionnaire

For the assessment of depression, the Patient Health Questionnaire (PHQ-9) is widely used [11]. However, a short version of the scale, such as the PHQ-2, is suitable for screening depression [12, 13]. In the PHQ-2, the participants were asked how often they experienced the two core symptoms of depressive disorder (i.e., “Little interest or pleasure in doing things” and “Feeling down, depressed, or hopeless”) over the past two weeks. Responses of the items were recorded on a 4-point Likert scale (0 = not at all, 1 = several days, 2 = more than half the days, 3 = nearly every day), where scores range from 0 to 6. A score of ≥ 3 was considered the cutoff point for depression. The Cronbach’s alpha was 0.86 in the present study.

#### Ethical consideration

This study was approved by the thesis committee at the Department of Public Health and Informatics, Jahangirnagar University, Dhaka, Bangladesh. For conducting the study, the Declaration of Helsinki 2013 was followed. The participants were informed about the study aims, benefits, or potential risks associated with participating in this study. No financial or other remuneration was given for participating in this study. All participants provided verbal and written consent before data collection.

#### Statistical analysis

Data was entered using Google Forms and prepared for formal analysis using SPSS Software. Frequency and percentages for the categorical variables and mean and standard deviation for the continuous variables were calculated. Data were normally distributed, and multicollinearity-related issues such as VIF (Variance Inflation Factor) and tolerance were absent. Chi-square test or t-test were performed to determine the association between the independent variables and depression. Significant variables in the univariate analysis were included in the logistic regression to identify the factors associated with depression among diabetic patients. The significant variables were included in the fitting of the model. Statistical significance was set as a two-tailed *p* < 0.05 for all the analyses.

### Systematic review and meta-analysis

#### Inclusion criteria

Inclusion criteria were (i) being an observational study (cross-sectional or case-control), (ii) conducted among diabetes patients in Bangladesh, (iii) reporting the prevalence of depression, (iv) being published in peer-reviewed journals, (v) using validated tools to assess depression, and (vi) being published in the English language.

#### Search strategy & study selection procedure

The present study adhered to the Preferred Reporting Items for Systematic Reviews and Meta-Analyses (PRISMA) guidelines [14]. PROSPERO registration ID number: 399766. A systematic literature search was conducted in *OVID*, *PubMed*, and *Web of Science* to include articles published by 3rd February 2023. Keywords such as depression, depressive symptoms, depressive disorder, diabetes, Bangladesh, and prevalence were combined with the Boolean operators (AND/OR/NOT) (details in the Supplementary file). All the keywords were based on the review question “What is the prevalence of depression among individuals with diabetes in Bangladesh?’

#### Data extraction

Data were extracted utilizing a pre-designed form with information containing the first author and publication year, study design, time frame, sample size and mean age, assessment tool and cutoff, prevalence rate, associated factors, prevalence assessment criteria, and quality assessment score. The entire process of this systematic review of methods of data extraction (Since confirming the search strategies, the study selection process, and the quality assessment) was completed independently by two reviewers (FAM & MMK). Any discrepancies between the two reviewers were resolved by a third reviewer (MAM) following a discussion with the two authors.

#### Quality assessment

The Newcastle Ottawa Scale (NOS) was used to assess the methodological quality of these studies. The three characteristics of selection, comparability, and outcome were investigated with the NOS. Three different versions of the checklist evaluate the cross-sectional (7 items) and case-control (8 items) studies. Each item is rated with one point except comparability (two points), with a maximum score of 9. A cutoff score of 5 detects a low risk of bias among the studies [15].

#### Statistical analysis

Random effect models were used for quantitative analysis, assuming within and between study variances [16]. The heterogeneity was estimated using the I^2^ statistic. I^2^ values of < 25%, 25–50%, 50% t 75%, and more than 75% represent mild, moderate, severe, and highly severe heterogeneity, respectively [17]. The publication bias was assessed using Egger’s and Begg’s tests and visual inspection of the funnel plots [18]. The Fill and Trim method served to identify missing studies due to publication bias [19]. To determine the sources of heterogeneity, sub-group analysis was conducted for the categorical variables (i.e., gender, study tool, cutoff of the assessment tools, study design) and univariate meta-regression for continuous variables (age, sample size, and quality assessment score). In addition, sensitivity analysis using the Jackknife method was used to evaluate each study’s effect on the pooled prevalence [20]. Furthermore, the inverse-variance method was used to estimate the pooled odds ratio. The pooled odds ratio was calculated if there was adequate information in at least four studies. The analysis was conducted using the STATA software version 17.

## Results

### Cross-sectional study

#### Description of the study participants

Data from 390 diabetic patients who completed the study were analyzed. The mean age of the participants was 51.81 ± 12.75 years (age range: 18–85). About 49.7% of participants were male, and most were married (87.7%), with 10.8% divorced/widow/separated and 1.5% single. About 53.2% lived in villages, 32.4% of participants reported having no formal education, most participants were housewives (43.6%), 62.7% of the participants reported suffering from other chronic diseases, 36.2% reported using tobacco, and 76.1% performed physical exercise daily for at least 30 min (Table 1). About 36.9% reported having a family history of diabetes, 60.8% reported taking medication as a treatment for diabetes, whereas 16.9% took both insulin and medication. The mean duration of diabetes was 7.89 ± 5.83 years, and there were 2.84 ± 2.46 visits to the doctor in the past six months (Table 2).

**Table 1:** Association between depression and socio-demographic variables

Variables	Total (*n*; %)	Yes *(n;* %)	χ^2^/t test value	*p*-value
**Age (Mean ± SD)**	51.81 ± 12.75	51.47 ± 13.21	0.311	0.756
**Gender**				
Male	194; 49.7%	37; 36.6%	9.370	**0.002**
Female	196; 50.3%	64; 63.4%		
**Marital status**				
Married	342; 87.7%	91; 90.1%	0.731	0.392
Others	48; 12.3%	10; 9.9%		
**Monthly family income (BDT)**				
0–15,000	157; 42.4%	26; 28%	13.916	**0.001**
15,001–30,000	104; 28.1%	27; 29%		
More than 30,000	109; 29.5%	40; 43%		
**Current residence**				
Village	207; 53.2%	57; 56.4%	0.569	0.451
City	182; 46.8%	44; 43.6%		
**Education**				
No education	125; 32.4%	17; 16.8%	19.581	**0.001**
Up to primary	36; 9.3%	15; 14.9%		
Up to secondary	116; 30.1%	39; 38.6%		
Up to higher secondary	48; 12.4%	15; 14.9%		
Graduation	43; 11.1%	12; 11.9%		
Post-graduation	18; 4.7%	3; 3%		
**Occupation**				
Service	56; 14.4%	13; 12.9%	22.007	**0.003**
Housewife	170; 43.6%	58; 57.4%		
Business	52; 13.3%	6; 5.9%		
Farmer	40; 10.3%	6; 5.9%		
Retired	33; 8.5%	10; 9.9%		
Student	4; 1.0%	-		
Day labor	22; 5.6%	2; 2%		
Others	13; 3.3%	6; 5.9%		
**Having chronic comorbidities/disease**				
Yes	244; 62.7%	70; 69.3%	2.528	0.112
No	145; 37.3%	31; 30.7%		
**Using tobacco product**				
Yes	141; 36.2%	35; 35%	0.091	0.763
No	248; 63.8%	65; 65%		
**Performing physical exercise**				
Yes	296; 76.1%	63; 62.4%	14.108	**< 0.001**
No	93; 23.9%	38; 37.6%

**Table 2:** Univariate associations between diabetes patient profile and depression

Variables	Total (*n*; %)	Yes *(n;* %)	χ^2^/t test value	*p*-value
**Family history of diabetes**				
Yes	144; 36.9%	46; 45.5%	4.350	**0.037**
No	246; 63.1%	55; 54.5%		
**Year of suffering from diabetes** (Mean ± SD)	7.89 ± 5.83	9.11 ± 6.64	-2.421	**0.016**
**Visiting a doctor in the past six months** (Mean ± SD)	2.84 ± 2.46	2.38 ± 2.47	2.155	**0.032**
**Treatment type**				
Medication	237; 60.8%	47; 46.5%	20.220	**< 0.001**
Insulin	52; 13.3%	15; 14.9%		
Both	66; 16.9%	31; 30.7%		
None	35; 9%	8; 7.9%		

### Prevalence of depression

Of the 390 participants, 101 scored ≥ 3 (out of 6) at the PHQ-2, such that the prevalence of depression in this cohort of diabetic patients was 25.9%.

#### Associations with depression

Gender showed a significant association with depression (χ^2^ = 9.370, *p* = 0.002). In addition, monthly family income (χ^2^ = 13.916, *p* = 0.001), education (χ^2^ = 19.581, *p* = 0.001), occupation (χ^2^ = 22.007, *p* = 0.003), and performing physical exercise (χ^2^ = 14.108, *p* < 0.001) were also significantly associated with depression (Table 1). A family history of diabetes showed a significant relationship with diabetes (χ^2^ = 4.350, *p* = 0.037). In addition, suffering from diabetes (χ^2^ = -2.421, *p* = 0.016), visiting a doctor in the past six months (χ^2^ = 2.155, *p* = 0.032), and treatment type (χ^2^ = 20.220, *p* < 0.001) were also significantly associated with depression among the participants (Table 2).

#### Multivariate model and potential predictors of depression in diabetic patients

After adjusting the variables that were significant in the univariate analysis, education, occupation, performing physical exercise, and treatment type emerged as independent predictors of depression among the 390 participants in this study. More specifically, those with only secondary education were at 4.63 times higher risk of depression than those with higher education (aOR = 4.63; 95% CI: 0.98–21.70, *p* = 0.052). Businessmen were less likely to have depression than others (aOR = 0.07; 95% CI: 0.01–0.40, *p* = 0.003). Similarly, people who were engaged in regular physical activity had a lower risk of depression than those who did not perform physical exercise for at least 30 min daily (aOR = 0.40; 95% CI: 0.21–0.77, *p* = 0.006). At the same time, those who were on both insulin and diabetic medications were approximately 5.73 times higher risk of depression (aOR = 5.73; 95% CI: 1.78–18.45, *p* = 0.003) (Table 3).

**Table 3:** Factors associated with depression in terms of sociodemographic and diabetes-related variables

Variables	aOR (95% CI)	*p*-value
**Gender**		
Male	1.59 (0.52–4.89)	0.411
Female	Ref.	
**Monthly family income (BDT)**		
0–15000	0.41 (0.17–1.03)	0.059
15001–30000	0.60 (0.29–1.25)	0.177
More than 30000	Ref.	
**Education**		
No education	1.34 (0.23–7.54)	0.740
Up to primary	3.99 (0.65–24.48)	0.134
Up to secondary	4.63 (0.98–21.70)	0.052
Up to higher secondary	3.62 (0.78–16.62)	0.098
Graduation	2.11 (0.45–9.95)	0.343
Post-graduation	Ref.	
**Occupation**		
Service	0.44 (0.09–1.98)	0.286
Housewife/homemaker	0.74 (0.16–3.39)	0.702
Business	0.07 (0.01–0.40)	0.003
Farmer	0.55 (0.09–3.16)	0.510
Retired	0.30 (0.05–1.66)	0.171
Student	-	-
Day labor	0.31 (0.03–2.70)	0.999
Others	Ref.	
**Performing physical activity > 30 min daily**		
Yes	0.40 (0.21–0.77)	0.006
No	Ref.	
**Family history of diabetes**		
Yes	1.54 (0.82–2.86)	0.172
No	Ref.	
**Year of suffering from diabetes** (Mean ± SD)	1.04 (0.99–1.10)	0.073
**Visiting a doctor in the past six months** (Mean ± SD)	0.92 (0.80–1.06)	0.260
**Treatment type**		
Medication	1.46 (0.54–3.94)	0.447
Insulin	2.52 (0.79–8.03)	0.118
Both	5.73 (1.78–18.45)	0.003
None	Ref.	

### Systematic review and meta-analysis

#### Description of the included studies

**Fig. 1:**
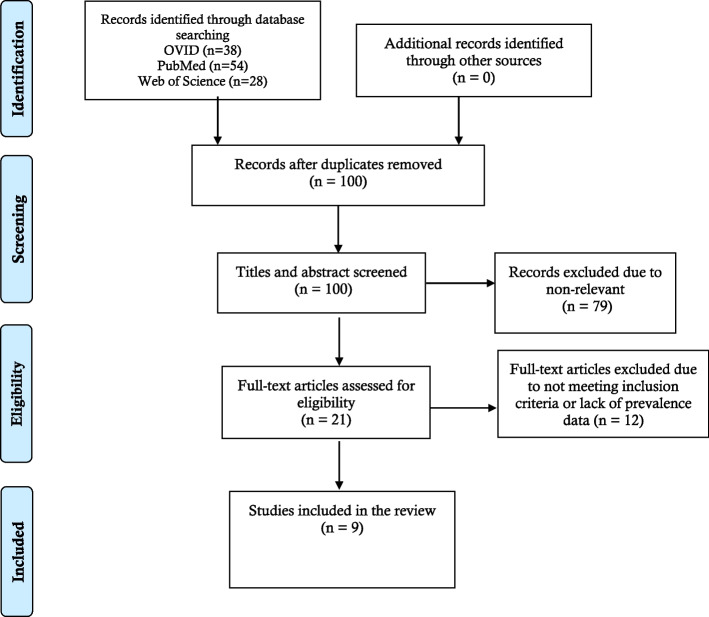
Prisma flow chat

After conducting a preliminary search, 120 articles were found across databases. Titles and abstracts of 100 retrieved articles were screened after excluding the duplicate articles. The duplicate articles were automatically removed by the Mendeley software (*n* = 20). Finally, nine articles were deemed suitable for the final analysis and abiding by the inclusion criteria. The details of the article screening procedure have been demonstrated in Fig. 1. In addition, the deleted full-text articles with reasons have been provided in the Supplementary file.

Of the included nine articles, the total number of diabetic patients was 4,025, with a mean age of 45.57 ± 10.49. Most were cross-sectional studies except for one case-control study. PHQ-9 was the most frequent tool (*n* = 6) to assess depression among diabetes patients, but two studies used the MADRS, while the HADS was used in one study. Among the studies, seven included both males and females, and the rest only had female participants. Two studies had participants with gestational diabetes, five with type-2 diabetes, and one did not report any specific type of diabetes. Detailed information regarding the study is presented in Table 4.

**Table 4:** Characteristics of the included articles in the review

First author & publication year	Study design	Time frame	Sample size (female %); mean age in years	Assessment tool; cutoff	Prevalence rate	Associated factors	Prevalence Assessment Criteria	Quality assessment score
Kamrul-Hasan et al., 2019 [21]	Cross-sectional	July 2017 to April 2018	900 (49.4%); 49.80 ± 11.50	PHQ-9; ≥ 5	60.3%	Family history of diabetes, comorbidities, diabetic complications, duration of diabetes, insulin use, insulin device, presence of albuminuria, chronic kidney disease.	Mild to severe	6
Islam, Rawal et al., 2015 [8]	Cross-sectional	September 2013 to July 2014	515 (55.9%); 50.2 ± 10.1	PHQ-9; ≥ 5	61.9%	Age, gender, number of complications, loss of business or crop failure, major family conflicts, separation or death of family members or divorce, unavailability of food or medicine	Minimal to severe	7
Islam, Ferrari et al., 2015 [22]	Case-control study	January to July 2014	Cases- 591 (56.9%); 51.4 ± 11.6	PHQ-9; ≥10	Case- 45.2%	NR	Mild to severe	5
Roy et al., 2012 [9]	Cross-sectional	November 2010 to February 2011	417 (49.4%); 53.2	PHQ-9; ≥10	16.5%	Age, gender, monthly income, residence, insulin and oral medication, cardiovascular disease, number of comorbidities, FBS level, HbA1c level.	Moderate to severe	6
Tasnim et al.,2022 [23]	Cross-sectional	January to December 2017	105 (100%); 28.98 ± 4.87	MADRS; ≥ 13	36.2%	History of reproductive health-related issues (i.e., abortion, neonatal death), uncontrolled glycemic status	Mild to severe	6
Sultana et al., 2022 [24]	Cross-sectional	May 5 to May 13, 2021	735 (54.3%); 52.30 ± 12.02	PHQ-9; ≥ 10	36.9%	Gender, marital status, education level, living status, chronic disorders, diabetes complications	Moderate to severe	8
Chowdhury et al., 2017 [25]	Cross-sectional	May 2012 to May 2013	121 (63.6%)	HADS; ≥8	56.2%	NR	Borderline abnormal to abnormal	5
Kamrul-Hasan et al., 2022 [10]	Cross-sectional	July 2019 and June 2020	259 (45.2%); 50.36 ± 12.7	PHQ-9; ≥ 10	40.5%	NR	Moderate to severe	7
Natasha et al., 2015 [26]	Cross-sectional	August 2011 to September 2012	382 (100%); 28.35 ± 5.34	MADRS; ≥ 13	25.92%	Age, dwelling place, history of gestational diabetes mellitus	Mild to severe	6

*PHQ-9* Patient Health Questionnaire-9, *MADRS* Montgomery Asberg Depression Rating Scale, *HADS* Hospital Anxiety and Depression Scale, *NR* Not reported

#### Prevalence of depression

**Fig. 2:**
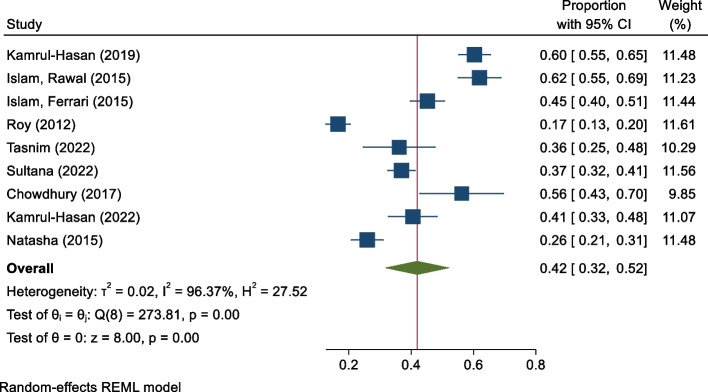
Prevalence of depression among diabetes patients in Bangladesh

Figure 2 provides the forest plot regarding the estimated pooled prevalence of depression. The pooled estimated prevalence of depression was 42% (95% CI: 32–52%, I^2^ = 96.37%) among the diabetes patients in Bangladesh.

#### Subgroup analysis

The subgroup analysis suggested that the prevalence of depression was 45% (95% CI: 33–57%, I^2^ = 96.85%) considering both genders (*n* = 7), whereas the rate was 30% (95% CI: 20–39%, I^2^ = 60.92%) for only female gender (*n* = 2) (χ^2^ = 3.78, *p* = 0.05) (Supplementary Fig. 1). For the study design, only one study was case-control, and the rest was cross-sectional (*n* = 8). The pooled prevalence of depression was 42% (95% CI: 30–53%, I^2^ = 96.72%) in the cross-sectional study (χ^2^ = 0.30, *p* = 0.58) (Supplementary Fig. 2). The pooled prevalence of mild to severe depression (*n* = 4) was 42% (95% CI: 27–57%, I^2^ = 95.74%), and the rate was 31% (95% CI: 16–46%, I^2^ = 96.12%) for moderate to severe depression (*n* = 3) (χ^2^ = 17.13, *p* < 0.001) (Supplementary Fig. 3). In addition, the prevalence was 43% (95% CI: 30–57%, I^2^ = 97.49%) when using the PHQ-9 scale (*n* = 6) (χ^2^ = 10.22, *p* = 0.01) (Supplementary Fig. 4). Based on subgroup analysis, the severity of depressive symptoms and the study tool accounted for the heterogeneity of the pooled prevalence.

#### Univariate meta-regression

Results from univariate meta-regression suggested that age (coefficient = 0.0042, standard error = 0.0059, *p* = 0.47) and sample size (coefficient = 0.00015, standard error = 0.00021, *p* = 0.47) had a positive association with depression, but a negative relationship was found between depression and quality assessment score (coefficient= -0.0104, standard error = 0.0609, *p* = 0.86). None of the associations was significant (*p* > 0.05).

#### Publication bias

**Fig. 3:**
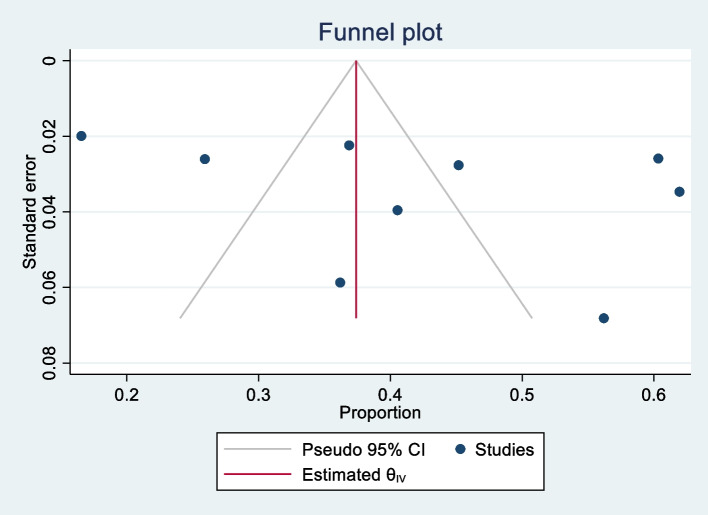
Funnel plot assessing publication bias among studies

Publication bias was assessed using the funnel plot and Egger’s test. Results showed no evidence of publication bias (Egger, *p* = 0.33; Begg, *p =* 0.46). The funnel plot is presented in Fig. 3.

#### Sensitivity analysis

**Fig. 4:**
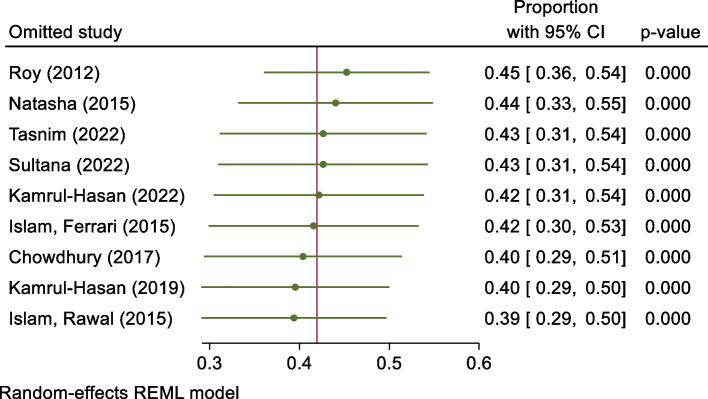
Study effects based on the Jackknife method

Sensitivity analysis was conducted based on the Jackknife method to determine each study’s effect. Results suggested that the pooled effect size was not affected by a single study effect (Fig. 4).

**Fig. 5:**
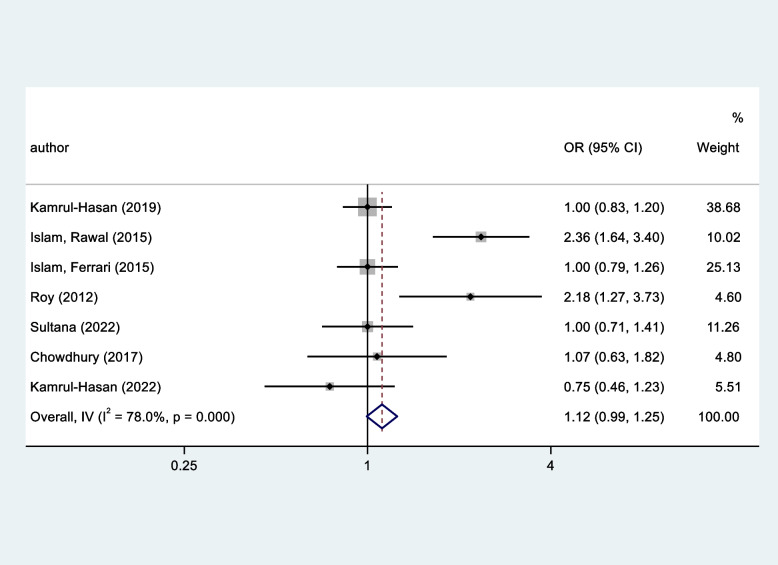
Relationship between gender and depression

#### Risk factors of depression

##### Socio-demographic factors

Gender had a significant association with depression. More specifically, females were at a 1.12-times higher risk of depression than males (OR = 1.12, 95% CI: 0.99 to 1.25, I^2^ = 78%, *p* < 0.001) (Fig. 5). People under 60 years of age exhibited a two-fold increased risk of depression than those > 60 years of age (S M S Islam et al., 2015a). However, one of the studies reported that higher age groups were at higher risk of depression [9]. Urban residents had a 1.3 times higher risk of depression than those living in rural settings [9]. In addition, a monthly income of less than 6000 BDT was associated with a high risk of depression compared to earning more than 6000 BDT [9]. A study reported that married or divorced/separated persons were less likely to be depressed than unmarried patients [24]. No formal education increased the risk of depression compared to a master’s or above degree [24]. In addition, patients living alone were at a 2.6 times higher risk of suffering from depression than those living with a spouse or children [24]. Suffering from chronic diseases such as hypertension, heart disease, etc., was reported significantly increase the risk of depression among patients in 3 studies [9, 21, 24]. Furthermore, a 2.2-fold higher risk was predicted among the patients experiencing major family conflicts [8].

##### Diabetes-related factors

a Diabetic related complications
  Patients with diabetic complications were at 3.15 times higher risk of depression than those without [21]. More specifically, having 13 diabetic co-morbidities and > 3 complications showed a 2.3-times and 2.1-times higher risk of depression, respectively [8]. Similarly, having diabetes-related difficulties increased the odds of depression by 2.17-fold [24].
b Insulin use and medication
  Insulin use also significantly increases the risk of diabetes among patients. Indeed, those patients requiring insulin had a 2.37 times higher risk of suffering from depression. In addition, albuminuria increased the risk of depression 4.04-fold [21]. Furthermore, the unavailability of food and medicine, higher level of FBS, and HbA1c levels also increased the risk of depression [8, 9].
c Others
  Diabetes mellitus patients experiencing diabetes for more than five years had an odd of 6.16 over those with less than five years of diabetes duration [21]. Similarly, a higher risk of depression was reported among patients with diabetes for more than ten years [24]. In addition, patients with a history of abortion and neonatal death had higher depression scores [23].

## Discussion

This study includes a cross-sectional assessment and a meta-analysis of relevant studies focused on Bangladesh. The major findings included a relatively elevated prevalence of depression and the identification of several risk factors contributing to depression risk. In particular, sex, age, educational level, occupation, physical activity, disease duration, and treatment type emerged as major risk factors. Thus, the present findings are anticipated to help policymakers develop screening and preventive measures to improve mental health well-being among diabetes patients in Bangladesh.

Based on the composite pooled prevalence, depression was present in 42% of Bangladeshi diabetic patients. Compared to data collected in other countries, a higher prevalence rate was reported in Iran (61.8%), whereby the pooled prevalence was 24.4% for mild, 19.1% for moderate, 11.4% for moderately severe, and 4.6% for highly severe among diabetes patients [27]. Conversely, a lower prevalence rate of depression was reported in Indian patients, 30% in young patients [28], and 38% across all ages [29]. These discrepant findings should not be viewed as conflictive but rather reflect the substantial heterogeneity of the epidemiology of diabetes from country to country and, of course, many other factors, such as access to healthcare. Even within the several studies retrieved from the systematic review and our cross-sectional study, a remarkable variance in the prevalence of depression was identified. It is, therefore, possible that the various risk factors identified herein as being independently associated with a higher prevalence of depression may be useful targets to address in healthcare campaigns aimed at reducing both the risk but also the overall adverse consequences of depression in a setting of underlying diabetes. The present meta-analysis found high heterogeneity in terms of reporting the pooled prevalence. It is likely because using different cutoff points and instruments to assess depression.

Of note, the factors associated with a higher risk of depression in the present study were very similar to those previously reported in both developed and developing economies [30–35]. Our cross-sectional study findings, which were confirmed to a great extent by the findings emanating from the meta-analysis, identified significant predictors of depression consisting of sex, age, education, occupation, physical activity, and treatment type. In a previous study, diabetic participants (mean age 40 years) had an increased occurrence of depression compared to participants without diabetes [36]. However, since the risk of depression seems to increase with age, reports of decreased risk at advanced age suggest different mediators potentially contributing to and modulating depression, notwithstanding the strong association between diabetes and depression [37]. Females were at higher risk of depression, as found in this study’s systematic review and meta-analysis. For example, studies reported that females had approximately 1.9 times the increased risk of depression than males [8]. Similarly, 2.8-times [9] and 1.8-times [24] risk of depression was predicted among the female gender by other studies. There is now robust evidence indicating that the female sex is another important risk factor for depression in diabetes. This observation has been consistently reported elsewhere and is further confirmed by the present study [27]. However, contradictory findings are also part of the extant literature whereby a study in India found that diabetic males were more likely to be depressed [38]. Since depression has also shown a profound association with the history of reproductive health-related issues (i.e., abortion, neonatal death), it should not be surprising that female diabetic patients more frequently than males report the presence of depressive symptoms [23].

Unemployment and low household income emerged as the most vulnerable groups to report depression [39]. Since diabetes incurs substantial healthcare costs, in a recent study, the perception of the unpredictability of clinical course and access to care in diabetes significantly mediated the relationship between living in poverty and glycosylated hemoglobin, while access to a healthy diet and lifestyle mediated the relationship between education level and diabetic control [40]. Furthermore, the authors further buttressed the observation that the significant effects of poverty and education attainment on glycosylated hemoglobin levels were mediated by avoidance coping and by depressive symptoms [40]. Additionally, taking medication for a long time and receiving insulin as treatment increased the odds of having depression, as reported in previous studies [36, 41] - similar findings have been reported elsewhere [42–44].

The study is not without limitations. First and foremost, due to the limited data details in each of the studies included in the meta-analysis, sex was the only risk factor that could be examined across all studies. Secondly, clinical trials and RCT-based studies were not included herein (as there were no studies conducted). However, this is the first systematic review and meta-analysis that aggregates the currently available evidence on depression among diabetes patients in Bangladesh, which also generates evidence from a cross-sectional survey. Finally, the generalizability of our findings to all regions of Bangladesh can only be assumed but was not specifically tested. Nevertheless, there is no particular reason to assume that beyond the heterogeneity factors described in the study, additional unidentified factors will be present and contribute substantially to the risk of depression in diabetic patients.

## Conclusions

In this study, we attempted to increase our understanding of the prevalence and factors associated with depression among Bangladeshi diabetic patients by implementing two distinct approaches, (i) cross-sectional study and (ii) systematic review and meta-analysis. The prevalence of depression, as per the cross-sectional data, was lower than the pooled prevalence of depression (25.9% vs. 42%), which may reflect the substantial heterogeneity in depression rates as dictated by multiple individual and societal elements acting as mediators of this relationship. The findings further revealed that diabetic females were at higher risk of depression.

Based on our findings, we would encourage policymakers to pay attention and integrate the predictors of depression among diabetic patients as identified by the cross-sectional data (i.e., education, occupation, physical activity, and treatment type), along with the predictors extracted from the systematic review (i.e., monthly income, location of residence, treatment type, family history of diabetes, comorbidities, diabetic complications, duration of diabetes, and others) to the treatment guidelines of diabetic patients in Bangladesh, such as to incorporate mental health screening and care.

## Supplementary Information

**Additional file 1.**

## Data Availability

The data supporting the present study’s findings are available from the corresponding author upon reasonable request.
