# Supplementary material for: Depression among Bangladeshi diabetic patients: a cross-sectional, systematic review, and meta-analysis study
Source: BMC Psychiatry. 2023 May 26;23:369. doi: 10.1186/s12888-023-04845-2 (PMC10214671; doi:10.1186/s12888-023-04845-2)
Supplement: Supplementary file 1 — Additional file 1. [file 12888_2023_4845_MOESM1_ESM.docx]

**Search strategy**

PubMed

("depressed"[All Fields] OR "depression"[MeSH Terms] OR "depression"[All Fields] OR "depressions"[All Fields] OR "depression s"[All Fields] OR "depressive disorder"[MeSH Terms] OR ("depressive"[All Fields] AND "disorder"[All Fields]) OR "depressive disorder"[All Fields] OR "depressivity"[All Fields] OR "depressive"[All Fields] OR "depressively"[All Fields] OR "depressiveness"[All Fields] OR "depressives"[All Fields] OR ("depression"[MeSH Terms] OR "depression"[All Fields] OR ("depressive"[All Fields] AND "symptoms"[All Fields]) OR "depressive symptoms"[All Fields]) OR ("depressive disorder"[MeSH Terms] OR ("depressive"[All Fields] AND "disorder"[All Fields]) OR "depressive disorder"[All Fields])) AND ("diabete"[All Fields] OR "diabetes mellitus"[MeSH Terms] OR ("diabetes"[All Fields] AND "mellitus"[All Fields]) OR "diabetes mellitus"[All Fields] OR "diabetes"[All Fields] OR "diabetes insipidus"[MeSH Terms] OR ("diabetes"[All Fields] AND "insipidus"[All Fields]) OR "diabetes insipidus"[All Fields] OR "diabetic"[All Fields] OR "diabetics"[All Fields] OR "diabets"[All Fields]) AND ("bangladesh"[MeSH Terms] OR "bangladesh"[All Fields] OR "bangladesh s"[All Fields]) AND ("epidemiology"[MeSH Subheading] OR "epidemiology"[All Fields] OR "prevalence"[All Fields] OR "prevalence"[MeSH Terms] OR "prevalance"[All Fields] OR "prevalences"[All Fields] OR "prevalence s"[All Fields] OR "prevalent"[All Fields] OR "prevalently"[All Fields] OR "prevalents"[All Fields])

WOS

1. (((TS=(depress*)) OR TS=("major depressive disorder")) OR TS=("depressive disorder"))
2. (((TS=(diabete*)) OR TS=("diabetes mellitus")) OR TS=("diabetes inspidus")) OR TS=("gestational diabetes")
3. (((AB=(prevalenc*)) OR AK=(prevalenc*)) OR TI=(prevalenc*)) OR ALL=(prevalenc*)
4. (((ALL=(Bangladesh*)) OR AD=(Bangladesh*)) OR TI=(Bangladesh*)) OR AK=(Bangladesh*)
5. 1 AND 2 AND 3 AND 4
6. 5 AND AND (DT==("ARTICLE") AND CU==("BANGLADESH"))

OVID

Embase <1974 to 2023 February 03>

APA PsycInfo <1806 to January Week 4 2023>

Ovid MEDLINE(R) and Epub Ahead of Print, In-Process, In-Data-Review & Other Non-Indexed Citations, Daily and Versions <1946 to February 03, 2023>

1 depress*.mp. [mp=ti, ab, hw, tn, ot, dm, mf, dv, kf, fx, dq, tc, id, tm, bt, nm, ox, px, rx, an, ui, sy] 1992361

2 "depress*".m_titl. 529002

3 1 or 2 1992361

4 diabet*.mp. [mp=ti, ab, hw, tn, ot, dm, mf, dv, kf, fx, dq, tc, id, tm, bt, nm, ox, px, rx, an, ui, sy] 2249084

5 "diabet*".m_titl. 960097

6 "diabetes mellitus".mp. [mp=ti, ab, hw, tn, ot, dm, mf, dv, kf, fx, dq, tc, id, tm, bt, nm, ox, px, rx, an, ui, sy] 1663663

7 "diabetes insipidus".mp. [mp=ti, ab, hw, tn, ot, dm, mf, dv, kf, fx, dq, tc, id, tm, bt, nm, ox, px, rx, an, ui, sy] 27919

8 "diabet*".m_titl. 960097

9 "diabetes mellitus".m_titl. 195811

10 "diabetes insipidus".m_titl. 9589

11 4 or 5 or 6 or 7 or 8 or 9 or 10 2249084

12 3 and 11 76409

13 prevalenc*.mp. [mp=ti, ab, hw, tn, ot, dm, mf, dv, kf, fx, dq, tc, id, tm, bt, nm, ox, px, rx, an, ui, sy] 2337489

14 "prevalenc*".m_titl. 405243

15 13 or 14 2337489

16 12 and 15 15127

17 bangladesh*.mp. [mp=ti, ab, hw, tn, ot, dm, mf, dv, kf, fx, dq, tc, id, tm, bt, nm, ox, px, rx, an, ui, sy] 52032

18 "bangladesh*".m_titl. 28439

19 17 or 18 52032

20 16 and 19 67

**Figure 1: Subgroup analysis of gender**

**Figure 2: Subgroup analysis of study design**

**Figure 3: Subgroup analysis of cutoff for the assessment tool**

**Figure 4: Subgroup analysis of used instrument**

**Table: Full-text articles deleted with reason**

| **Title** | **Reason for excluding** |
| --- | --- |
| - Assessment of health-related quality of life of Bangladeshi patients with type 2 diabetes using the EQ-5D: a cross-sectional study - Health‐related quality of life and its predictors among the type 2 diabetes population of Bangladesh: A nation‐wide cross‐sectional study - Non-adherence to self-care practices & medication and health related quality of life among patients with type 2 diabetes: a cross-sectional study - The relationship between medical comorbidities and health-related quality of life among adults with type 2 diabetes: The experience of different hospitals in southern Bangladesh - Association between comorbidity and health-related quality of life in a hypertensive population: a hospital-based study in Bangladesh | Reported combine prevalence of depression/anxiety |
| - Temporal changes in the prevalence of depression and diabetes mellitus in a rural population of Bangladesh: from 2004 to 2009 - Diabetes and depression among pregnant women in Bangladesh: a hospital based study - Pregnancy outcome in depressive and GDM subjects in Bangladesh: a hospital based comparative study | Only abstract is available/poster presentation |
| - Health-related quality of life among people with type 2 diabetes mellitus - A multicentre study in Bangladesh - Prevalence of Type 2 Diabetes and Impaired Glucose Regulation with Associated Cardiometabolic Risk Factors and Depression in an Urbanizing Rural Community in Bangladesh: A Population-Based Cross-Sectional Study - Prevalence of depression and diabetes: a population-based study from rural Bangladesh - Prevalence of Depression and Glucose Abnormality in an Urbanizing Rural Population of Bangladesh | Did not report the prevalence of depression among diabetic patients |
|  |  |
